# Mediating effect of amygdala activity on response to fear vs. happiness in youth with significant levels of irritability and disruptive mood and behavior disorders

**DOI:** 10.3389/fnbeh.2023.1204574

**Published:** 2023-10-12

**Authors:** Ji-Woo Suk, Robert J. R. Blair, Brigette Vaughan, Arica Lerdahl, William F. Garvey, Ryan Edwards, Ellen Leibenluft, Soonjo Hwang

**Affiliations:** ^1^Department of Psychiatry, University of Nebraska Medical Center, Omaha, NE, United States; ^2^Digital Health Research Division, Korea Institute of Oriental Medicine, Daejeon, Republic of Korea; ^3^Child and Adolescent Mental Health Centre, Mental Health Services, Capital Region of Denmark, Emotion and Development Branch, Copenhagen, Denmark; ^4^National Institute of Mental Health, Bethesda, MD, United States

**Keywords:** irritability, mediator, amygdala, disruptive mood and behavior disorder, emotion, facial expression, fMRI

## Abstract

**Introduction:**

Irritability, characterized by a tendency to exhibit increased anger, is a common clinical problem in youth. Irritability is a significant clinical issue in youth with various psychiatric diagnoses, especially disruptive behavior, and mood disorders (Attention-Deficit/Hyperactivity Disorder, Oppositional Defiant Disorder, Conduct Disorder, and Disruptive Mood Dysregulation Disorder). Although there have been previous studies focusing on functional alteration in the amygdala related to irritability, there is no comprehensive model between emotional, neuronal, and behavioral characteristics.

**Methods:**

Using an functional magnetic resonance imaging (fMRI) procedure, we investigated the relationships between behavioral irritability, selective impairments in processing facial emotions and the amygdala neural response in youth with increased irritability. Fifty-nine youth with disruptive mood and behavior disorder completed a facial expression processing task with an event-related fMRI paradigm. The severity of irritability was evaluated using the Affective Reactivity Index.

**Results:**

In the result of behavioral data, irritability, and reaction time (RT) differences between interpreting negative (fear) and positive (happiness) facial expressions were positively correlated. In the fMRI result, youth showed higher activation in the right cingulate gyrus, bilateral cerebellum, right amygdala, right precuneus, right superior frontal gyrus, right middle occipital gyrus, and middle temporal gyrus, during the happiness condition vs. fear condition. No brain region exhibited greater activation in the fear than in the happiness conditions. In the result of the mediator analysis, increased irritability was associated with a longer RT toward positive vs. negative facial expressions. Irritability was also positively associated with the difference in amygdala blood oxygen level-dependent responses between the two emotional conditions (happiness > fear). This difference in amygdala activity mediated the interaction between irritability and the RT difference between negative and positive facial expressions.

**Discussion:**

We suggest that impairment in the implicit processing of facial emotional expressions with different valences causes distinct patterns of amygdala response, which correlate with the level of irritability. These results broaden our understanding of the biological mechanism of irritability at the neural level and provide information for the future direction of the study.

## 1. Introduction

Irritability in youth is defined as a low threshold to frustration and increased proneness to anger, relative to peers (Leibenluft, 2017a). It is one of the most common reasons youth are referred for mental health treatment (Avenevoli et al., 2015). Due to its clinical significance, Disruptive Mood Dysregulation Disorder (DMDD) in which irritability is the cardinal symptom was added to the Diagnostic and Statistical Manual of Mental Disorders, fifth edition (DSM-5) (American Psychiatric Association, 2013). Severe irritability is one of the main psychopathologies in youth with disruptive mood and behavior disorders (DMBD), including Attention Deficit Hyperactivity Disorder (ADHD) (Karalunas et al., 2014), Oppositional Defiant Disorder (ODD) (Herzhoff and Tackett, 2016), Conduct Disorder (CD) (Euler et al., 2015), and Disruptive Mood Dysregulation Disorder (DMDD) (Dougherty et al., 2014).

One potential mechanism of irritability in youth concerns impairment in the processing of others’ emotional states (Siever, 2008; Bilgi et al., 2017). The rapid and accurate processing of emotions in human faces is a basic neuropsychological ability central to emotion regulation (Shaw et al., 2014). Impaired processing of facial expressions can lead to abnormal reactions, exacerbating dysfunctional emotion regulation and social interactions (Legenbauer et al., 2016). Several studies have examined the relationship between the process of emotional face and psychosocial impairments in youth with severe levels of irritability (Guyer et al., 2007; Rich et al., 2008). In these studies, youth with severe irritability showed deficient processing of various subsets of emotions including surprise (Rich et al., 2008), anxiety (Buhle et al., 2014), happiness (Guyer et al., 2007; Buhle et al., 2014), anger (Guyer et al., 2007; Kim et al., 2013), sadness (Guyer et al., 2007), fear (Guyer et al., 2007), and disgust (Buhle et al., 2014) compared to healthy youth, and these deficits are associated with significant psychosocial impairments (Rich et al., 2008).

In addition, studies reported that youth with severe irritability demonstrated biases in emotion processing toward a specific direction (Hommer et al., 2014; Stoddard et al., 2016). Youth with irritability have a bias toward threat cues (Hommer et al., 2014), and label an ambiguous face as angry compared to healthy youth, suggesting that youth with irritability may perceive neutral faces as threatening, which may result in reactive aggressive behavior (such as anger outbursts) (Hommer et al., 2014; Stoddard et al., 2016). However, it is noteworthy that a recent controlled study (Haller et al., 2022) did not support a positive effect of computerized recognition training for ambiguous faces on improving irritability in 22 children and adolescents with DMDD. These findings, although in a small number of patients, suggested that various other features (i.e., behavioral deficits, neural correlates, and phenotypic specificity) should be considered in a comprehensive model of underlying neurobiological mechanisms in youth with irritability. Additionally, aberrant threat interpretation may be considered as one cognitive pathway to emotional problems across internalizing and externalizing phenotypes of which irritability can be a critical part (Haller et al., 2022). In this study, we therefore aim to identify how the behavioral phenotype (severity of irritability) and its neurological correlates influence the aberrant threat processing in youth with DMBD.

In regard to the neural areas, studies demonstrated that aberrant processing of emotional faces in youth with irritability was related to altered activation in the neural areas such as the amygdala, middle frontal cortex, medial frontal cortex, and anterior cingulate cortex (Hommer et al., 2014; Stoddard et al., 2016; Crum et al., 2020; Kryza-Lacombe et al., 2020). Furthermore, increased irritability levels were associated with altered right amygdala connectivity to the left superior frontal gyrus when viewing negatively interpreted facial expressions (Kryza-Lacombe et al., 2020). However, until now, most studies exploring neural areas involved in the processing of facial expressions in youth with irritability focused on the expression of negative emotions (such as fear and anger) (Thomas et al., 2013; Hommer et al., 2014; Stoddard et al., 2016; Kircanski et al., 2018). Therefore, little is known about the differentiated neural responses between positive and negative facial emotion processing in youth with irritability.

In this regard, one previous study (Wiggins et al., 2016) showed that compared to healthy youth, youth with irritability showed increased amygdala activation to positive valence faces (happy faces at 75% intensity), but decreased amygdala activation to negative valence faces (fearful faces at 50% intensity and angry faces at 75% intensity). In another study (Brotman et al., 2010), youth with irritability showed hypoactivation of the amygdala to negative facial expressions compared to healthy youth and youth with ADHD. However, both studies focused on comparisons among youth with severe irritability (Severe Mood Dysregulation), healthy youth, and youth with other categorical diagnoses (ADHD or Bipolar Disorder). Thus, further investigation is warranted to determine the role of emotion-processing regions (including the amygdala) in the interaction between the processing of facial emotions of *various valences* and irritability *within* a group of youth with a severe level of irritability.

We set three goals in this study: We sought to determine (1) the relationship between irritability in youth and selective impairment in the processing of negatively (fear) versus positively (happiness) interpreted facial expression; (2) whether differences in the neural response toward negative (fearful) versus positive (happy) facial expressions are related to irritability; and (3) whether the amygdala response mediates the interaction between irritability and the differentiated behavioral response (reaction time, RT) toward negative (fear) vs. positive (happiness) facial expression. Participants performed an implicit facial expression processing task (Habel et al., 2007; Quarto et al., 2014), observing faces with different degrees of fear and happiness intensity during functional magnetic resonance imaging (fMRI) scanning. We predicted (1) a positive relationship between irritability and impaired processing of negative (fearful) relative to positive (happy) facial expressions (Guyer et al., 2007); (2) a positive relationship between irritability and amygdala activation toward negative (fearful) vs. positive (happy) facial expressions (Wiggins et al., 2016; Kryza-Lacombe et al., 2020); and (3) Amygdala responses to emotions would mediate relationships between the processing of emotions (indicated by behavioral data) and irritability.

## 2. Materials and methods

### 2.1. Participants

Participants were recruited from the outpatient clinic of a large academic medical center in the Midwest and its surrounding community. A structured interview was conducted using the Kiddie-Schedule for Affective Disorders and Schizophrenia for School-Age Children-Present and Lifetime Version (K-SADS) (Kaufman et al., 1997) by a licensed and board-certified child and adolescent psychiatrist and/or advanced practice psychiatric nurse to confirm their psychiatric diagnoses.

The inclusion criteria were: (1) aged 10–18 years, (2) a clinically significant level of irritability as defined by a score of 4 or greater (≥ 4) on the self-reported Affective Reactivity Index (ARI) (Stringaris et al., 2012), (3) no cognitive disability and intelligent quotient (IQ) > 70 measured by the Wechsler Abbreviated Scale of Intelligence, Second Edition (2-subtest form) (Wechsler, 2011), (4) no past or present history of neurological disease, (5) no comorbid psychotics, tic, or substance abuse disorders, (6) no primary diagnosis of autism spectrum disorder with significant impairments in communication and significant behavioral disturbance, (7) no use of anxiolytics (benzodiazepines), and (8) no metal in the body, claustrophobia, or other condition that would preclude MRI. Psychotropic medication use (stimulants, alpha-agonists, antipsychotics, antidepressants, and mood stabilizers) was not exclusion if the dose and schedule had been consistent for at least 6 weeks.

Fifty-nine youth completed the fMRI experiment. Thirteen youth were excluded from the analysis [i.e., missing ARI questionnaires (*n* = 2), technical difficulties of the MRI scanner (*n* = 6), and head motion exceeding 2 mm (*n* = 5)].

All participants and their guardians provided written informed consent. Youth were provided of the assent and gave their signed approval. The study was approved by the University of Nebraska Medical Center Institutional Review Boards (IRB # 321-16-FB) at the participating academic medical center. All methods and procedures in this study were in accordance with the current guidelines of the World Medical Associations Declaration of Helsinki.

### 2.2. Measures

Participants and their guardians provided demographic information such as age, sex, ethnicity, and clinical information associated with irritability and behavioral problems.

The severity of irritability was measured using the parent- and self-report forms of the ARI (Stringaris et al., 2012). The ARI has good reliability with a test–retest correlation coefficient of 0.80 and Cronbach’s alpha of 0. 92 (Stringaris et al., 2012; Mulraney et al., 2014). The ARI has 7 items screening the child’s irritable behavior and impairment over the past 6 months on a 3-point Likert scale. To mitigate reporter bias (Fisher and Katz, 2000), the total irritability score was generated using the summation of parent- and self-report ARI scores in the analysis.

The Behavioral Assessment Scales for Children (BASC) (version 2) is a screening tool for assessing internalizing and externalizing behavioral problems in children and adolescents based on the parent’s self-report (Reynolds and Kamphaus, 2015; Dowdy et al., 2019; Tan et al., 2020). The BASC is composed of 12 subscales, four composite scales, nine content scales, and four executive functioning indices with each item utilizing a 4-point Likert-type scale (“Never” to “Almost Always”) (Reynolds and Kamphaus, 2015). Test–retest reliabilities have been reported to range from 0.81 to.92, and internal consistency has ranged from 0.83 to 0.95 (Reynolds and Kamphaus, 2015; Dowdy et al., 2019; Tan et al., 2020).

### 2.3. fMRI task

Participants completed a facial expression processing task with an event-related fMRI paradigm (Marsh et al., 2008). The detailed description of the fMRI task is available in Supplementary data.

### 2.4. Data acquisition

Neuroimaging data were collected using a 3.0-Tesla Siemens Skyra MRI scanner. A detailed explanation of data acquisition is available in Supplementary data.

### 2.5. Behavioral data analysis

Mean reaction time (RT) and percent accuracy (ACC) were calculated in each emotional condition. To normalize its distribution, RT was transformed using the following equation: log (1/RT) (Whelan, 2008).

The repeated measure Analysis of variance (ANOVA) was performed to compare the behavioral data between conditions (i.e., happy and fearful conditions) using the log-transformed RT. Since the transformed ACC was not normally distributed, the ACC between conditions was analyzed non-parametrically using the Friedman test. All analyses were conducted using SPSS version 26.0 (IBM Corp., Armonk, NY, United States).

To examine the relationship between irritability and the differences in behavioral responses (i.e., ACC and RT) between fear and happiness, correlation analyses were conducted with the ARI score and the differences (i.e., subtracting ACC and RT of fear condition from those of happiness condition).

### 2.6. fMRI analysis

SPM12^1^ implemented in Matlab R2017a (MathWorks) was utilized for preprocessing and statistical analysis. To ensure steady-state magnetization, the first three volumes were discarded. After the correction of slice acquisition timing, each of the 238 echo planar images (EPI) volumes was realigned to the first functional image using an affine (six parameters) spatial transformation to correct for head movements during the fMRI acquisition. The time series volumes were then co-registered with the high-resolution anatomical image and normalization to the standard brain of the Montreal Neurological Institute was performed. The spatially normalized EPI volumes were smoothed by an 8-mm fullwidth-half-maximum Gaussian kernel.

A design matrix for the emotional (i.e., happiness and fear) and baseline (i.e., fixation) conditions was created using a box-car function convolved with the canonical hemodynamic response function and its temporal derivative. To reduce the residual temporal fluctuation of EPI volumes, the general linear model included the six movement parameters of rigid body transformation applied by the realignment procedure as nuisance variables. Also, high-pass filtering was applied using a discrete cosine transform set with a cutoff frequency of 1/128 Hz in the design matrix. To identify the neural substrates related to the processing of happy and fearful facial expressions, statistical parametric maps of the *t*-statistic (SPM{*t*}) were created for each participant and the contrast images were archived.

Paired t-tests of the general linear model (GLM) beta weight coefficients were then performed between the functional activities of the happy condition and the fear condition. To examine whether the difference of neural response between the two emotions is related to irritability, correlation analysis was conducted between the ARI score and beta value differences of happiness vs. fear. The threshold is set to voxel-wise *p* < 0.001 uncorrected and cluster *p* < 0.05 corrected with 10 contiguous voxels (Eklund et al., 2016).

### 2.7. Mediation analysis

A mediation analysis was conducted to examine whether the differential blood oxygen level-dependent (BOLD) responses in the amygdala in response to fearful vs. happy faces mediate the association between irritability and the differences in RT in response to fearful vs. happy faces. The beta values were extracted from an amygdala (i.e., region of interest, ROI) based on the fear vs. happiness contrast image for each participant. The amygdala ROI was created by placing a 5-mm sphere around the coordinates in Table 1. We hypothesized that the effect of irritability on the differences in RT between negative (fearful) and positive (happy) facial expressions would be mediated by the differential BOLD responses in the amygdala between the two conditions. We tested mediation using the Sobel test (Baron and Kenny, 1986) accompanied by a bootstrapping method with *N* = 5,000 bootstrap samples (Preacher and Hayes, 2004) using the PROCESS macro procedure (Hayes, 2012) for SPSS version 26 (IBM). The model was estimated through Process Model 4 (Darlington and Hayes, 2017). PROCESS reports bias-corrected 95% confidence intervals as indicators of significance.

**Table 1:** Comparison of fMRI activation to fear and happiness.

Area	Side	Cluster	*Z*-value	*x*	*y*	*z*
Happiness > Fear						
Cingulate gyrus	R	168	4.73	6	−20	36
Cerebellum IV, V, VII	L	170	4.35	−8	−54	−4
Amygdala	R	114	4.19	30	2	−16
Precuneus	R	58	3.94	12	−60	54
Superior frontal gyrus	R	44	3.91	10	32	62
Middle occipital gyrus	R	67	3.93	44	−66	−10
Middle temporal gyrus	R	61	3.78	48	−48	0
Cerebellum IV, V, VII	R	70	3.78	14	−62	−26

L, left hemisphere; R, right hemisphere.

## 3. Results

### 3.1. Participant characteristics

A total of 46 youth (73.91% male) participated in this study [mean age: 13.72, Standard Deviation (SD) = 2.34 years, IQ: 99.91 (SD = 14.09)]. The prevalence of mental disorders was: DMDD (50%), ADHD (73.91%), ODD (58.70%), CD (13.04%), and depressive disorders (21.74%) (see Table 2).

**Table 2:** Demographic and clinical characteristics.

Variable	Category	Value
Gender, N (%)	Boy	34 (73.91)
	Girl	12 (26.09)
Age, mean (SD)	–	13.76 (2.31)
Age, N (%)	Older than 6 ~ under 11	4 (8.70)
	Older than 11 ~ under 14	17 (36.95)
	Older than 14 ~ under 18	23 (50)
	Older than 18	2 (4.35)
Diagnosis, N (%)	ADHD	34 (73.91)
	ODD	27 (58.70)
	CD	6 (13.04)
	DMDD	23 (50.00)
	MDD	10 (21.74)
Cognitive intelligence score, mean (SD)	–	99.91 (14.09)
BASC scale, mean (SD)	Hyperactivity	61.36 (11.89)
	Aggression	60.42 (11.53)
	Conduct problem	62.36 (13.15)
ARI scale, mean (SD)	Total	14.24 (4.18)
	ARI-S	6.13 (2.13)
	ARI-P	8.11 (2.61)
fMRI task accuracy, %, mean (SD)	Neutral condition	93.87 (0.14)
	Fearful condition	93.52 (0.14)
	Happy condition	93.82 (0.14)
fMRI task RT, ms, mean (SD)	Neutral condition	910.06 (123.94)
	Fearful condition	923.03 (122.16)
	Happy condition	924.74 (122.78)

ADHD, Attention Deficit Hyperactivity Disorder; ARI-S, Self-report Affective Reactivity Index; ARI-P, Parent-report Affective Reactivity Index; BASC, Behavioral Assessment Scales for Children; CD, Conduct Disorder; DMDD, Disruptive mood dysregulation disorder; MDD, Major Depression Disorder; ODD, Oppositional Defiant Disorder; RT, reaction time.

Irritability severity measured by ARI (i.e., the sum of parent- and self-report scores) was 14.24 (SD = 4.18). Behavioral problems as measured by BASC-2 were as follows: 61.36 (SD = 11.89) of hyperactivity, 60.42 (SD = 11.53) of aggression, and 62.36 (SD = 13.15) of conduct problems (see Table 2).

### 3.2. Results of the behavioral data

A repeated measure ANOVA on log-transformed RT showed no difference between negative and positive valence facial emotions (*F* = 1.88, *p* = 0.16). Also, the Friedman test did not reveal any difference of ACC between negatively and positively interpreted facial expressions (X^2^ = 1.29, *p* = 0.53). The -mean values of RT and ACC -for each emotional condition are shown in Table 2.

However, there was a positive correlation between irritability and the RT difference between happiness and fear (*r* = 0.445, *p* < 0.01). There was no significant correlation between irritability and the ACC difference (*r* = 0.211, *p* = 0.159).

### 3.3. fMRI results

In the fear and happiness vs. baseline conditions, activation was higher in the bilateral pre- and supplementary motor area [Brodmann area, (BA) 4, 6], bilateral visual cortex (BA 18, 19), bilateral insula (BA 13), right middle and inferior frontal gyrus (BA 46, 9), right putamen, left anterior cingulate gyrus, right parietal lobe (BA 40), right caudate and right thalamus (for all, *p* < 0.05, FDR corrected).

Paired *t*-test revealed that youth showed higher activation in the right cingulate gyrus (BA 23), bilateral cerebellum, right amygdala, right precuneus (BA 7), right superior frontal gyrus (BA 8), right middle occipital gyrus (BA 37), and middle temporal gyrus (BA 37), during the happiness condition vs. fear condition. No brain region exhibited greater activation in the fear than in the happiness conditions (Table 1).

**Figure 1:**
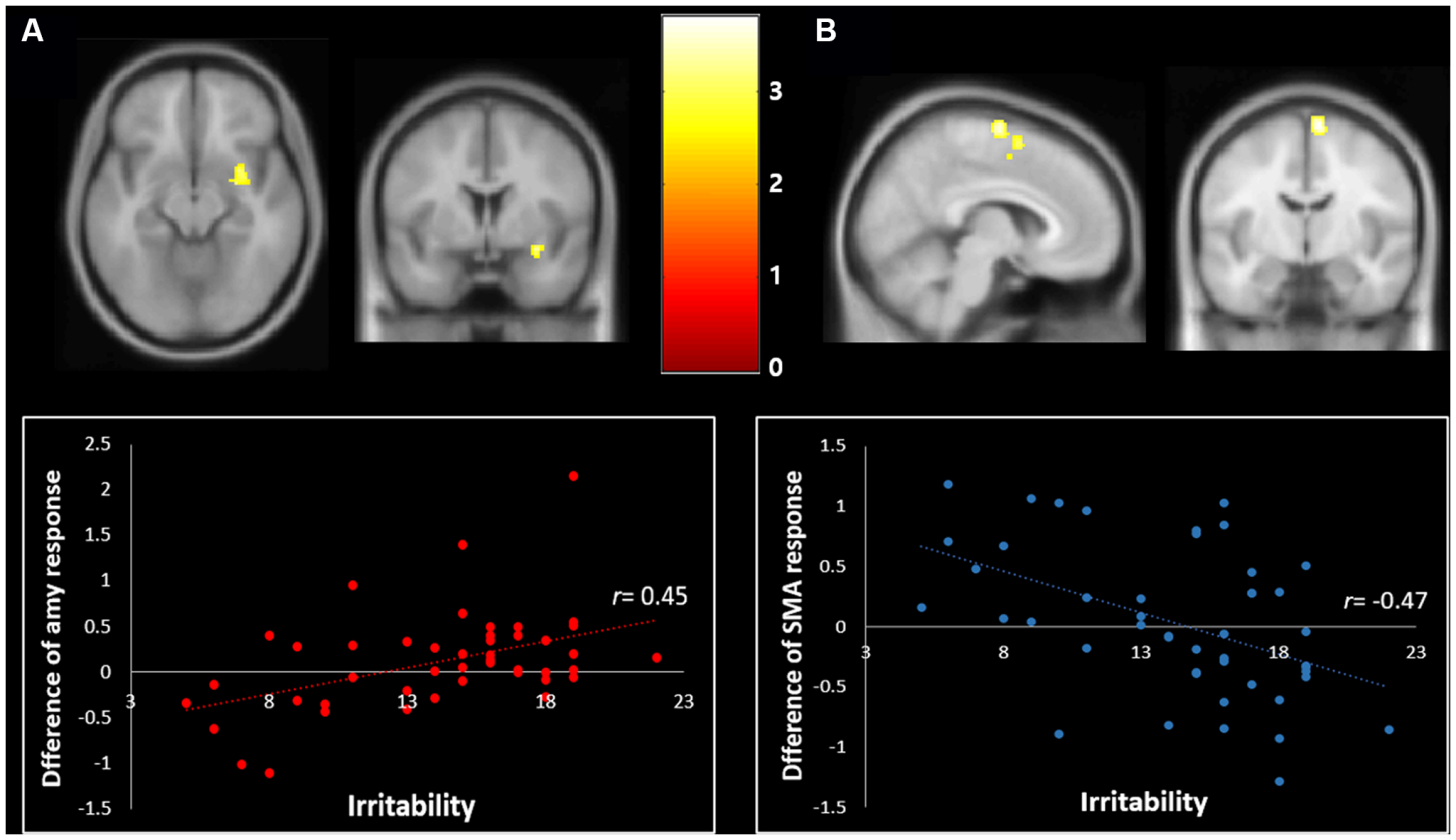
Correlation of irritability with the differential neural response toward fear vs. happiness (*p* < 0.001, uncorrected, *k* = 50). **(A)** The severity of irritability was positively correlated with the differential BOLD response changes in response to the happy relative to fearful facial expressions in the right amygdala (Talairach coordinate: 30, −6, −12) (*r* = 0.45, *p* < 0.01). **(B)** The severity of irritability was negatively correlated with the differential BOLD response changes of the right supplementary motor area in response to the happiness relative to the fearful facial expressions (Talairach coordinate: 7, −8, 70) (*r* = −0.47, *p* < 0.01).

Correlation analyses to identify brain regions associated with the severity of irritability (i.e., ARI score) indicated the right amygdala (Z score of maximum = 3.91; Talairach coordinate: 30, −6, −12) and right supplementary motor area (Z score = 3.78; Talairach coordinate: 7, −8, 70) (*p* < 0.001, uncorrected, k = 50) during happiness than fear conditions. Post-hoc analyses revealed that irritability severity was significantly correlated with the difference of percent signal changes during the happiness compared to fear conditions in the right amygdala (*r* = 0.45, *p* < 0.01) and supplementary motor area (*r* = −0.47, *p* < 0.01), as shown in Figure 1.

### 3.4. Results of mediator analysis

**Figure 2:**
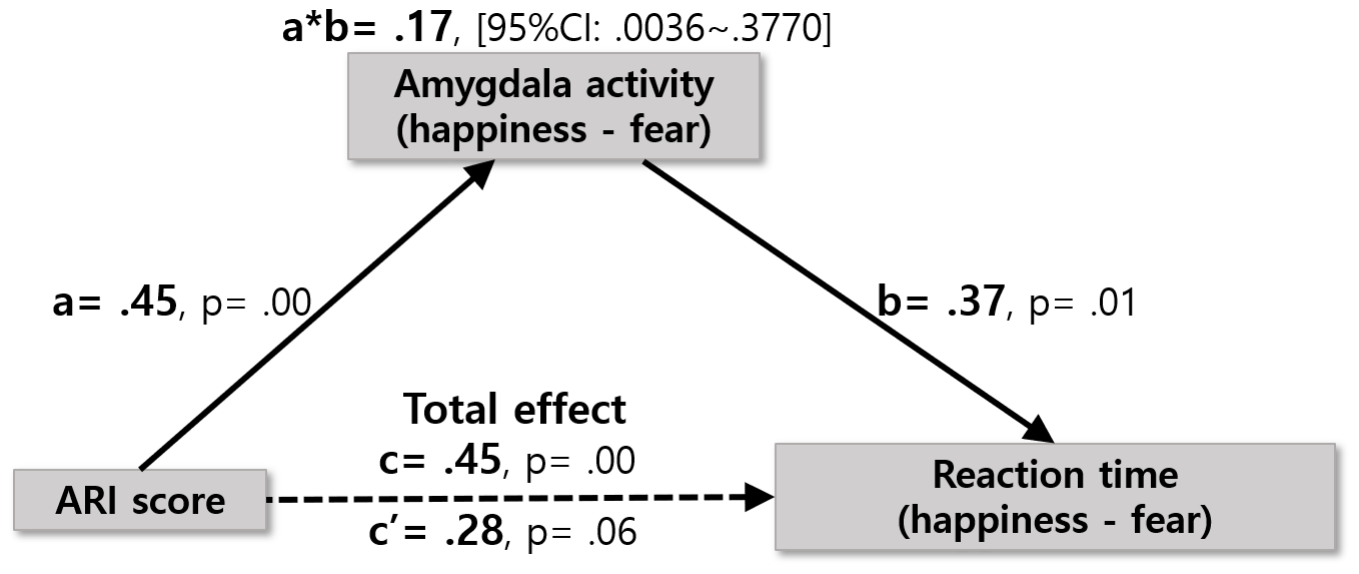
Full mediation model. Standardized beta coefficients for amygdala activity mediating effect on the relationship between ARI score and RT. The amygdala activity mediated the influences of the ARI score and reaction time (indirect effect = 0.17; 95% CI: 0.0036–0.3770). The total effect (c) of ARI score (summation of youth ARI and parent ARI) and reaction time was significant (c = 0.45, *p* = 0.00), but after taking the significant mediation effect of amygdala activity into consideration, the remaining direct effect (c’) of reaction time was reduced and no longer significant (c’ = 0.28, *p* = 0.06).

The mediation analysis demonstrated that the association between irritability and the difference in RT between negative (fear) and positive (happiness) facial expressions was fully mediated through the difference in amygdala activity between the two conditions, as shown in Figure 2. Tables 3, 4 describe the direct and indirect effects. Separate regression models were estimated for each path.

**Table 3:** Mediating effects of the difference of amygdala response between fear and happiness on the difference of reaction time between fear and happiness.

IV	DV: Difference of amygdala response between fear and happiness			DV: Difference of reaction time between fear and happiness		
	β	*SE*	*t*	β	*SE*	*t*
Constant	0.00	0.13	0.00	0.00	0.13	0.00*
ARI	0.45	0.14	3.30**	0.28	0.14	1.97
Difference of amygdala responses between fear and happiness				.37	0.14	2.56*
	*R*^2^ = 0.20, *F* = 11.29, *p* = 0.002			*R*^2^ = 0.30, *F* = 9.42, *p* = 0.000		

*p < 0.05, **p < 0.01, ***p < 0.001. IV, independent variable; DV, dependent variable; ARI, Affective Reactivity Index.

**Table 4:** Bootstrapping result of mediating effects.

Variables	Indirect effect	Boot SE	95% CI	
			LLCI	ULCI
Difference of amygdala response between fear and happiness	02.1651	0.0966	0.0036	0.3770

Boot LLCI, the lower limit of the 95% confidence intervals; Boot ULCI, the upper limit of the 95% confidence intervals.

Firstly, the difference in amygdala responses between the two emotions was regressed on irritability. The standardized coefficient (β) is 0.45 (95% CI = 0.18–0.72), indicating that the difference in amygdala activity strongly predicted irritability (*p* < 0.01). Similarly, the difference of RTs between the two emotions was regressed on the difference in amygdala responses (standardized coefficient = 0.37, *p* < 0.05, 95% CI = 0.14–0.78). However, there is no direct effect of irritability on the RT difference between the two emotions (standardized coefficient = 0.28, *p* = 0.06, 95% CI = −0.01 to 0.57). Next, bootstrapping was performed to determine the statistical significance of the indirect effect. As a result, the total effect (ab + c’) is 0.28 (β), and the indirect effect via mediator (ab) is 0.17 (β). Since 0 was not contained in the 95% confidence interval (CI = 0.00–0.38), the indirect effect of amygdala activity significantly mediated the relationship between the ARI score and the difference in RT. Hence, it supports our hypothesis that the difference between two emotions in amygdala activity mediates between the severity of irritability and the difference in RT between two emotions.

## 4. Discussion

The aim of this study using an implicit emotion processing task (Habel et al., 2007; Quarto et al., 2014) was to examine (1) the relationship between irritability and selective impairment of specific facial expression processing in behavioral response [indicated by reaction time (RT)]; (2) the relationship between irritability and the difference in neural responses to negative (fearful) and positive (happy) facial expressions; and (3) the mediation effect of amygdala activity on the relationship between irritability and selective impairment in the facial expression processing of various valences (fear vs. happiness).

There were three main findings. First, we found a positive relationship between irritability and the difference in RT between negative (fearful) and positive (happy) facial expressions. The more irritable youth were, the longer the RT to the happy face expression compared to the fearful face expression. Second, the paired comparison between negative and positive facial expressions in youth showed engagement of the right cingulate gyrus, bilateral cerebellum, right amygdala, right precuneus, right superior frontal gyrus, right middle occipital gyrus, and middle temporal gyrus. In all these areas, youth showed increased activation in response to positive vs. negative emotional expressions. Specifically, irritability levels were significantly correlated with an increased differential blood-oxygen-level-dependent imaging (BOLD) response changes to happiness relative to fear processing in the amygdala (i.e., positive correlation) and supplementary motor area (i.e., negative correlation). Lastly, the difference in amygdala activity between fear and happiness conditions completely mediated the interaction between irritability and the RT difference between negative and positive facial expressions.

As we predicted, greater irritability was associated with longer RT for gender selection when a happy face was presented relative to a fearful face. Additionally, there was a negative correlation between irritability level and RT in response to the fearful faces (*r* = −0.30, *p* < 0.05), but no significant association between irritability and RT to the happy faces (*r* = −0.10, *p* = 0.50) (Supplementary Figure S1). Reaction time is an indicator of the speed of unobservable mental processes and has been widely used as a measure of the associative strength between stimulus and response (Leppänen et al., 2003; Kimura et al., 2012). Specifically, longer RT indicates high implicit attentional capture (i.e., emotional interference) by some categories of stimuli in an implicit emotion processing task (McKenna and Sharma, 1995; Arioli et al., 2021), suggesting that the shorter RT in the fear condition may be associated with impaired processing of the fear emotions. This potentially means the greater the irritability in youth, the less emotional interference in the fear condition there was, suggesting a deficit of fear processing that was positively correlated with irritability in these youth.

Previous studies reported that irritability was associated with impaired processing of fear emotion in youth at the neural level with severe mood dysregulation, bipolar, depressive, anxiety disorders, or schizophrenia (Shankman et al., 2013; Vidal-Ribas et al., 2016; Bilgi et al., 2017). However, when behavioral data were examined, there was little evidence supporting the relationship between irritability and impaired processing of fear in DMBD (i.e., DMDD, bipolar or severe mood dysregulation disorder), even though there were differences in neuroimaging findings (Brotman et al., 2010; Wiggins et al., 2016). The neuroimaging study with explicit emotion task (i.e., face emotion labeling) reported the association between irritability and dysfunctional activation in the amygdala and other temporal and prefrontal regions in the DMBD group but failed to elicit the relationship between irritability and behavioral response, suggesting that neuroimaging data may have captured more subtle neural level differences than behavioral response.

Besides the lower detection power of behavioral data, another potential reason for the non-detection of the significant results in behavioral data might be a lack of consideration for individual differences of RTs to stimuli with various emotional valences. The previous studies did not control this nuisance variable by only focusing on the mean difference among categorical groups (for example, youth with severe mood dysregulation vs. youth with bipolar disorder) or the relationship between RT and irritability in a single emotion. To overcome this, we assessed the time difference between two types of emotions with opposite valences *within* an individual, which made it possible to examine the impairment of specific emotions within the study group.

As for the neuroimaging results, greater activation was found in the right posterior cingulate gyrus (BA 23), bilateral cerebellum, right amygdala, right precuneus (BA 7), right superior frontal gyrus (BA 8), right middle occipital gyrus (BA 37), and middle temporal gyrus (BA 37) in response to happiness relative to fear. No area showed increased activation of the fear relative to the happiness condition, suggesting that at the neural level, the processing load increases in the happiness condition more than the fear one. Previous studies have delineated an extensive network involved in face processing in humans, including the fusiform gyrus (BA 37) involved in facial recognition, the frontal eye field (BA 8) participating in gaze shift control, and precuneus in processing visuospatial information and drawing attention to the external environment, and several limbic system regions such as the amygdala for recognizing emotional expressions and posterior cingulate cortex for emotional salience/mediating interactions between emotion and memory (Kanwisher et al., 1997; Haxby et al., 2000; Ishai et al., 2000; Shah et al., 2001).

In line with behavioral data, the neuroimaging result demonstrated that facial expression with negative valences elicits decreased activation of these core neural areas relative to facial expressions with positive valences in youth with irritability. Previous studies also showed similar results (Brotman et al., 2010; Thomas et al., 2013; Wiggins et al., 2016) but the current study expands these previous ones by elucidating the differentiated neural responses in these areas toward facial expressions with negative versus positive valence *within* a group of youth with severe irritability. These findings may suggest that youth with irritability might have specific dysfunctions in the processing of emotional faces, which might have clinical implications.

Specifically, the role of the right amygdala in the neurobiology of irritability was further supported by its full mediation of the relationship between the difference of responses (the differences of RT between positive and negative valences) and end-point symptom manifestation (irritability level). Previous work on the visual processing of emotional facial expressions, especially fearful face processing, has revealed the involvement of the right amygdala when faces are presented subliminally to prevent conscious detection (Cecere et al., 2014; Troiani et al., 2014; Framorando et al., 2021). For this reason, researchers have focused on examining the role of amygdala dysfunction in the neural underpinnings of irritability in youth (Vidal-Ribas et al., 2016; Wiggins et al., 2016). In the previous studies, youth with DMBD showed decreased activity in the amygdala during the processing of fearful facial emotion processing relative to healthy controls (Brotman et al., 2010; Wiggins et al., 2016). Our study furthers these previous findings by showing a positive relationship between irritability and the different activity of the right amygdala in fear vs. happiness, suggesting the higher the irritability, the more likely there is an impaired function in the amygdala specifically in response to negative emotion processing.

In this regard, it requires further assessment of how irritability and the amygdala are interactively linked to the differentiated processing of various emotions. Wiggins et al. (2016) demonstrated that amygdala response differed significantly depending on emotion and irritability level (Copeland et al., 2013); youth in the DMBD group showed greater levels of irritability with greater activation in response to happy and anger emotions, but less activation with increasing irritability to the fear (Wiggins et al., 2016). This study suggests the interaction effect of the amygdala and irritability on specific emotions. Our result further demonstrated that the amygdala is a full mediator in the interaction between irritability and the behavioral data (i.e., RT) difference between fear and happiness conditions. This could be interpreted as higher irritability prompts a greater difference in amygdala activity between happiness and fear, which in turn reflects a greater difference in facial emotion processing between the two emotions.

Only one area that showed a negative correlation between irritability level and differences of BOLD responses between positive and negative valences was the pre-supplementary motor area (pre-SMA). The pre-SMA, a part of the premotor cortex (BA 4 and 6), serves functions of motor control or top-down cognitive control including conflict monitoring, error detection, response selection, and attention control (Obeso et al., 2013). The previous neuroimaging studies showed the relationship between irritability and functional and structural impairment in the pre-SMA, suggesting that the pre-SMA might be critical for the neurobiology of irritability (Fishburn et al., 2019; Seok et al., 2021). In line with these studies, our result revealed that the higher irritability, the less RT occurred in the fear compared to the happiness condition because of a relatively lower influence of emotional interruptions.

Even though many previous studies showed significant correlations between irritability in youth and impaired amygdala function during the process of negative emotional stimuli, the direction of the amygdala dysfunction (hypo- vs. hyperactivation) has varied across different studies (Brotman et al., 2010; Thomas et al., 2012, 2013; Wiggins et al., 2016). The difference in the results might be due to the following factors: (1) the nature of task paradigms aiming at implicit versus explicit emotional processing; (2) Stimuli design including various valences of emotional stimuli (negative only versus comparison between negative and positive); (3) target population (especially various categorical diagnoses); (4) potentially differentiated neural mechanisms of irritability mediated by other clinical phenotypes (for example, various degrees of callous-unemotional trait); (5) low effect size driven by a small subset of the sample (Lieberman et al., 2007; Anticevic et al., 2012; Leibenluft, 2017b; Gu et al., 2019; Lee et al., 2022).

Specifically, previous evidence suggested the amygdala response may vary depending on the type of task (especially implicit vs. explicit) (Lieberman et al., 2007). Implicit face-processing tasks may activate the amygdala more reliably relative to explicit processing tasks which require the higher-order cognitive processing involved in emotional labeling and engage the association cortex such as the prefrontal cortex (Lieberman et al., 2007).

It is also noteworthy that most studies that reported amygdala hyperactivation during the processing of negatively interpreted faces focused on the threat cues (such as anger) (Coccaro et al., 2007; Beaver et al., 2008; Carré et al., 2012). Even though anger and fear are categorized as unpleasant emotions (i.e., negative valence) and have a similar arousal level, their hedonic direction (approach versus avoidance) is the opposite (Posner et al., 2005; Gu et al., 2019).

Additionally, to examine one of the most important confounding factors (i.e., callous-unemotional trait), especially in the presence of hypo-activation of the amygdala to negative valence facial expression (Lozier et al., 2014), we performed the mediator analysis by using the callous-unemotional trait as a covariate. In this analysis, we still found the mediating effect of the amygdala on the correlation between irritability and the selective fear impairment indicated by the behavioral data (Supplementary Tables S1, S2). Also, the effect of irritability on the difference in amygdala response between two emotions (standardized coefficient = 0.36) was significantly greater than that of the callous-unemotional trait (standardized coefficient = 0.28), suggesting that irritability was more involved in emotion-specific deficit in the amygdala than the callous-unemotional trait, at least in our study group.

Taken together, the role of impairment in the emotion processing areas (such as the amygdala) in the development of irritability in youth requires further studies to determine the specific relation between various types of emotions and the implicated neural areas.

We have a few caveats to offer. First, this is a cross-sectional study and cannot be applied to the longitudinal and neurodevelopmental aspects of irritability in youth. Second, our task implicitly evaluated fear and happiness processing to identify the relationship between irritability and lack of emotion-specific processing. This limits the generalization of our findings into other aspects of emotion processing and its relation to irritability, such as explicit facial expression recognition, top-down attention control of facial emotional expression, or other kinds of emotional stimuli (for example, sadness or anger). Further research is therefore warranted. Third, the sample size of youth with severe irritability (*n* = 46) was relatively small for mediation analysis, although the results were statistically robust. Also, on the other hand, the sample we used in this study had the advantage of obtaining data from youth with psychiatric diagnoses established by a structured interview using K-SADS-PL (Kaufman et al., 2000). Many participants were on psychotropic medications (91.52%%) although the medication doses and schedule had to be stable at least 6 weeks before enrollment. Thus, the level of irritability as well as the neural responses in the emotional responding/emotion regulation areas were equally under the influence of participants’ current medications. However, a larger sample study will be required in the future to completely rule out the effect of psychotropic medications on this mediation. Lastly, to define the irritability for the inclusion criteria, a self-report ARI score was applied. The validity of the self-report scale is vulnerable to social desirability bias (Fisher and Katz, 2000). To mitigate purported validity problems, the summation of parent and self-report ARI scores was applied to create a total irritability score in the analysis. Also, the Pearson correlation value between the two scores was 0.79 (*p* < 0.001).

Despite these limitations, our findings extend previous research regarding the neurobiology of irritability in youth. We demonstrated impairment in the implicit process of facial emotional expression in distinct patterns of amygdala activation that corresponded to varying levels of irritability. Our findings, in particular, support the use of an irritability specifier for the diagnosis of children with DMBD, as well as the increased sensitivity and power that result from interpreting clinical measurements as dimensional rather than dichotomous/categorical variables (Beauchaine and Tackett, 2020; Bell et al., 2021). In children with varied levels of irritability, the current findings link observable patterns of emotional recognition with specific patterns of neural dysfunction. These findings highlight the necessity of taking temperamental and personality factors into account, as well as behavior patterns when developing successful, personalized treatment plans for youth with irritability.

## Data availability statement

The original contributions presented in the study are included in the article/Supplementary material, further inquiries can be directed to the corresponding author.

## Ethics statement

The studies involving humans were approved by the University of Nebraska Medical Center Institutional Review Boards (IRB # 321-16-FB). The studies were conducted in accordance with the local legislation and institutional requirements. Written informed consent for participation in this study was provided by the participants’ legal guardians/next of kin.

## Author contributions

RB, SH, and J-WS: study conception and design. BV, AL, WG, and RE: data collection. RB, SH, J-WS, and EL: interpretation of results. SH and J-WS: draft manuscript preparation. All authors reviewed the results and approved the final version of the manuscript.

## Footnotes

^1^ http://www.fil.ion.ucl.ac.uk/spm

## Abbreviations

ACC: Accuracy
ADHD: Attention Deficit Hyperactivity Disorder
ARI: Affective Reactivity Index
BA: Brodmann Area
BASC: Behavioral Assessment Scales for Children
CD: Conduct Disorder
DMBD: Disruptive Mood and Behavior Disorder
DMDD: Disruptive Mood Dysregulation Disorder
EPI: Echo Planar Images
fMRI: functional Magnetic Resonance Imaging
K-SADS: Kiddie-Schedule for Affective Disorders and Schizophrenia for School-Age Children-Present and Lifetime Version
ODD: Oppositional Defiant Disorder
pre-SMA: pre-supplementary motor area
RT: Reaction Time
