# Supplementary material for: Mediating effect of amygdala activity on response to fear vs. happiness in youth with significant levels of irritability and disruptive mood and behavior disorders
Source: Front Behav Neurosci. 2023 Oct 12;17:1204574. doi: 10.3389/fnbeh.2023.1204574 (PMC10602729; doi:10.3389/fnbeh.2023.1204574)
Supplement: Supplementary file 1 [file Data_Sheet_1.docx]

***fMRI task***

Participants completed a facial expression processing task with an event-related fMRI paradigm (Marsh *et al.*, 2008). Participants were shown a picture of the emotional expressions of 10 men and women from the Pictures of Facial Affect series (Ekman, 1976). The expressions were fearful (i.e., negative valence) or happy (i.e., positive valence). The emotional faces showed the parametrically modulated intensity of each emotional expression (25%, 50%, 100%, and 150% intensity). For emotional faces, in addition to standard (100%) expression intensity, lower (50%) and higher (150%) expression intensities were created by morphing with the neutral expression picture. A parametric design was used for ecological validity, as people usually experience varying levels of face expression intensity (Marsh *et al.*, 2008). Participants labeled the gender of the faces using two response buttons. Faces were presented for 2500 msec followed by a 500-msec fixation cross. There are four runs in the task and each of the four runs involved 80 face trials (10 trials of each intensity of fearful and happy faces) and 25 fixation trials. The trial order was randomized within each run. Total duration of the task is approximately 10 min.

***Data acquisition***

Neuroimaging data were collected using a 3.0-Tesla Siemens Skyra MRI scanner. A total of 152 functional images were taken with a gradient echo planar imaging (EPI) sequence: repetition time = 2500 ms; echo time = 27 ms; 64 × 64 matrix; 90° flip angle; 24 cm field of view. Images were acquired in 46 slices of 2.5 mm with 0.5 mm spacing per brain volume.

A T1-weighted magnetization-prepared rapid gradient-echo sequence was acquired to aid with spatial normalization: 176 axial slices, repetition time (TR) = 2200 ms; echo time (TE) = 2.48 ms; flip angle = 8°; field of view (FOV) = 23 × 23 cm2; matrix = 256 × 208; slice thickness = 1 mm; voxel size = 0.9 × 0.9 × 1 mm^3^.

Supplementary Figure 1. Correlation of irritability with reaction times of each condition. a) fear condition (r= -0.30, p< .05), b) happiness condition (r= -0.10, p= .50)


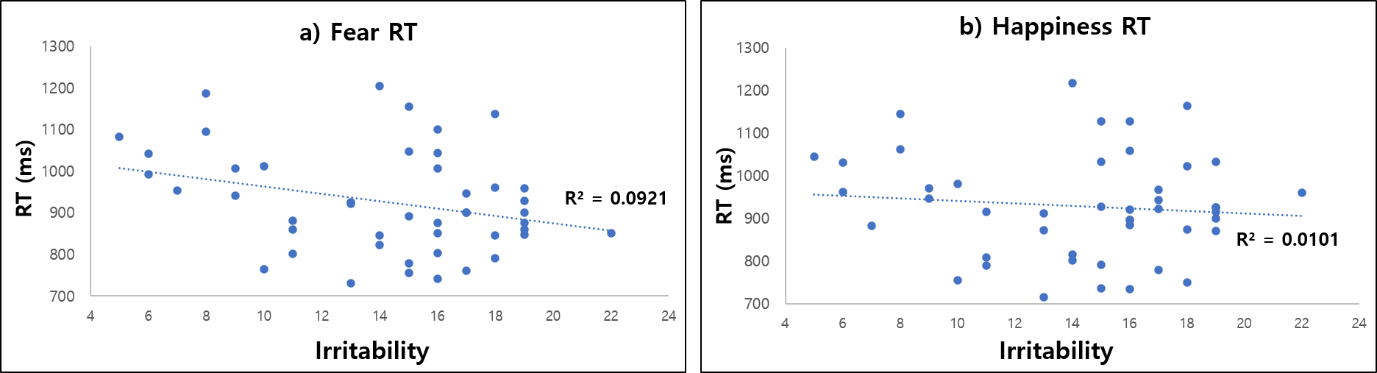


Supplementary Table 1. Mediating effects of amygdala on the relationship between irritability and emotion recognition after eliminating the effect of callous-unemotional trait.

| **IV** | **DV: Difference of amygdala response between fear and happiness** | | | **DV: Difference of reaction time between fear and happiness** | | |
| --- | --- | --- | --- | --- | --- | --- |
|  | ***B*** | ***SE*** | ***t*** | ***B*** | ***SE*** | ***t*** |
| **Constant** | -1.14 | .33 | -3.51** | -51.73 | 32.55 | -2.06 |
| **ARI** | .05 | .02 | 2.68* | 3.16 | 1.67 | 1.89 |
| **ICU** | .01 | .00 | 2.08* | .07 | .43 | .17 |
| **Difference of amygdala responses between fear and happiness** |  |  |  | 31.74 | 12.65 | 2.37* |
|  | *R^2^*= .28, F= 8.22, *p*= .000 | | | *R^2^*= .31, F= 6.15, *p*= .000 | | |

*p<.05, **p<.01, ***p<.001

IV, Independent variable; DV, dependent variable; ARI, Affective Reactivity Index; ICU, Inventory of callous-unemotional traits

Supplementary Table 2. Bootstrapping result of mediating effects after eliminating the effect of callous-unemotional trait.

| **Variables** | **Indirect Effect** | **Boot SE** | **95% CI** | |
| --- | --- | --- | --- | --- |
|  |  |  | **LLCI** | **ULCI** |
| **Difference of amygdala response between fear and happiness** | 1.49 | .95 | -.03 | 3.55 |

Boot LLCI : The lower limit of the 95% confidence intervals

Boot ULCI : : The upper limit of the 95% confidence intervals

***Reference***

Dowdy, E., DiStefano, C., Greer, F., Moore, S. & Pompey, K. (2019). Examining the latent structure of the BASC-3 BESS Parent Preschool Form. Journal of Psychoeducational Assessment 37, 181-193.

Eklund, A., Nichols, T. E. & Knutsson, H. (2016). Cluster failure: Why fMRI inferences for spatial extent have inflated false-positive rates. Proceedings of the national academy of sciences 113, 7900-7905.

Ekman, P. (1976). Pictures of facial affect. consulting psychologists press.

Marsh, A. A., Finger, E. C., Mitchell, D. G., Reid, M. E., Sims, C., Kosson, D. S., Towbin, K. E., Leibenluft, E., Pine, D. S. & Blair, R. (2008). Reduced amygdala response to fearful expressions in children and adolescents with callous-unemotional traits and disruptive behavior disorders. American Journal of Psychiatry 165, 712-720.

Mulraney, M. A., Melvin, G. A. & Tonge, B. J. (2014). Psychometric properties of the Affective Reactivity Index in Australian adults and adolescents. Psychological assessment 26, 148.

Reynolds, C. & Kamphaus, R. (2015). Behaviour assessment system for children–third edition manual. Circle Pines, MN: American Guidance Service.

Stringaris, A., Goodman, R., Ferdinando, S., Razdan, V., Muhrer, E., Leibenluft, E. & Brotman, M. A. (2012). The Affective Reactivity Index: a concise irritability scale for clinical and research settings. Journal of Child Psychology and Psychiatry 53, 1109-1117.

Tan, T. X., Yi, Z., Kamphaus, R. W., Wang, Y., Li, Z. & Cheng, K. (2020). Testing the reliability and construct validity of a Chinese translation of BASC-3-SRP for 12-to 18-year-old youth. Journal of Psychoeducational Assessment 38, 599-612.
